# Editorial: Current Concepts in Elbow Trauma

**DOI:** 10.2174/1874325001711011345

**Published:** 2017-11-30

**Authors:** Chetan S. Modi

**Affiliations:** Consultant Shoulder and Elbow Surgeon, University Hospitals Coventry and Warwickshire NHS Trust, UK

Elbow injuries are relatively common and have a significant socioeconomic impact, accounting for 10% of all injuries to the upper limb with almost one third of these being dislocations and approximately 10% being fractures [1, 2]. Furthermore, the elbow is the second most commonly dislocated joint in adults and the most commonly dislocated joint in the paediatric population with 40% of these injuries being associated with sport [2, 3]. Injuries patterns can range from simple low-energy dislocations up to high-energy complex fractures and fracture-dislocations.

Historically, the outcomes of complex elbow injuries have been poor with complications including stiffness, recurrent instability, malunions, heterotopic ossification and post-traumatic arthritis [4, 5]. More recently, a better understanding of the relevant osseous and ligamentous anatomy of the elbow with structured step-wise protocols has resulted in better outcomes from even the most severe injuries [6].

The goal of treatment is to restore the elbow to a stable joint with a pain-free functional range-of-motion. Most activities can be performed with a flexion/ extension arc of 30-130 degrees, 50 degrees of pronation and 50 degrees of supination [7]. To achieve this, the treating specialist requires an in-depth knowledge of the anatomy, injury patterns and available operative techniques around the elbow to correctly reconstruct both the osseous and ligamentous components of the injury. The purpose of this special issue is to provide the reader with a comprehensive overview of the most up-to-date techniques and evidence regarding the assessment and management all aspects of common elbow trauma.
